# Structural studies demonstrating a bacteriophage-like replication cycle of the eukaryote-infecting *Paramecium bursaria* chlorella virus-1

**DOI:** 10.1371/journal.ppat.1006562

**Published:** 2017-08-29

**Authors:** Elad Milrot, Eyal Shimoni, Tali Dadosh, Katya Rechav, Tamar Unger, James L. Van Etten, Abraham Minsky

**Affiliations:** 1 Department of Structural Biology, The Weizmann Institute of Science, Rehovot, Israel; 2 Chemical Research Support, The Weizmann Institute of Science, Rehovot, Israel; 3 Proteomics, The Weizmann Institute of Science, Rehovot, Israel; 4 Department of Plant Pathology and Nebraska Center for Virology, University of Nebraska, Lincoln, NE, United States of America; Institut Pasteur, FRANCE

## Abstract

A fundamental stage in viral infection is the internalization of viral genomes in host cells. Although extensively studied, the mechanisms and factors responsible for the genome internalization process remain poorly understood. Here we report our observations, derived from diverse imaging methods on genome internalization of the large dsDNA *Paramecium bursaria* chlorella virus-1 *(*PBCV-1). Our studies reveal that early infection stages of this eukaryotic-infecting virus occurs by a bacteriophage-like pathway, whereby PBCV-1 generates a hole in the host cell wall and ejects its dsDNA genome in a linear, base-pair-by-base-pair process, through a membrane tunnel generated by the fusion of the virus internal membrane with the host membrane. Furthermore, our results imply that PBCV-1 DNA condensation that occurs shortly after infection probably plays a role in genome internalization, as hypothesized for the infection of some bacteriophages. The subsequent perforation of the host photosynthetic membranes presumably enables trafficking of viral genomes towards host nuclei. Previous studies established that at late infection stages PBCV-1 generates cytoplasmic organelles, termed viral factories, where viral assembly takes place, a feature characteristic of many large dsDNA viruses that infect eukaryotic organisms. PBCV-1 thus appears to combine a bacteriophage-like mechanism during early infection stages with a eukaryotic-like infection pathway in its late replication cycle.

## Author summary

Although extensively studied, the mechanisms responsible for internalization of viral genomes into their host cells remain unclear. A particularly interesting case of genome release and internalization is provided by the large *Paramecium bursaria* chlorella virus-1 (PBCV-1), which infects unicellular eukaryotic photosynthetic chlorella cells. In order to release its long dsDNA genome and to enable its translocation to the host nucleus, PBCV-1 must overcome multiple hurdles, including a thick host cell wall and multilayered chloroplast membranes that surround the host cytoplasm. Our observations indicate that these obstacles are dealt with perforations of the host wall, the host cellular membrane, and the host photosynthetic membranes by viral-encoded proteins. Furthermore, our results highlight a bacteriophage-like nature of early PBCV-1 infection stages, thus implying that this virus uniquely combines bacteriophage-like and eukaryotic-like pathways to accomplish its replication cycle.

## Introduction

A fundamental and general stage in viral infection is the transfer of the viral genome into the host cell. After attachment to the cell membrane, viruses that infect animal cells depend on various entry pathways, mainly consisting of endocytosis, pinocytosis, phagocytosis and variants of these strategies [1,2]. Thus, un-coating of viral genomes occurs inside the host cell. In contrast, most bacteriophages eject their genome into their bacterial host through the cell wall and membrane layers [3,4], eventually leaving an empty capsid at the periphery of the bacterial cell.

*Paramecium bursaria* chlorella virus-1 (PBCV-1) is the prototype of the genus *Chlorovirus* (family *Phycodnaviridae*) that infects chlorella-like green algae and along with viruses in the *Mimiviridae*, *Asfarviridae*, *Poxviridae*, *Iridoviridae* and Marseilleviridae families, is a member of the nucleocytoplasmic large eukaryote-infecting dsDNA viruses clade [5,6]. Viruses belonging to this clade have recently attracted interest due to their unusual size, structural complexity, large genomes and elaborate infection cycles [7,8].

PBCV-1 is an icosahedral virion (190 nm in diameter) that, like bacteriophages, needs to penetrate a thick host cell wall and cellular membranes to initiate infection [9,10]. The virus contains a single spike-like structure at one vertex [11], which makes the first contact with the wall of its host cell [12], the unicellular photosynthetic alga *Chlorella variabilis* NC64A. PBCV-1 attachment is followed by host cell wall degradation at the point of contact by a virus-packaged enzyme(s) [9]. As reported here, following wall degradation the viral internal membrane fuses with the host membrane, thus generating a membrane-lined tunnel through which the ~331kbp linear dsDNA viral genome and viral proteins are ejected into the host cytoplasm [10], leaving an empty viral capsid on the cell surface [9], a trait characteristic of bacteriophages. Once ejected, the viral genome is rapidly translocated to the host nucleus, as indicated by the finding that transcription of viral genes is detected in infected cells at 7 min post infection (PI) [13]. This finding, along with the fact that the virus neither encodes nor packages a recognizable RNA polymerase support the notion that at least initial viral DNA replication and transcription processes occur in the host nuclei. This notion is also consistent with recent observations revealing major morphological modifications of the host nucleus during PBCV-1 infection [14]. Indeed, no extensive morphological changes of host nuclei are detected during the replication cycle of the giant Mimivirus or the Vaccinia virus whose entire replication cycles take place in the cytoplasm [15,16].

These observations raise several fundamental questions. The large internal pressure generated by the highly condensed genome in bacteriophages, along with pull forces exerted by bacterial DNA-binding proteins such as RNA polymerases present in the cytoplasm, have been suggested to contribute to viral DNA ejection [4,17–21]. Neither of these factors can account for the ejection of the PBCV-1 genome, as the pressure generated by the PBCV-1 genome, although substantial, is significantly less than that characteristic of bacteriophages [22], and no DNA-binding proteins are expected to be present in chlorella cytoplasm. Thus, what are the mechanisms responsible for PBCV-1 genome ejection? Moreover, PBCV-1 genomes ejected into the host cytoplasm are rapidly translocated to the host nucleus [23]. The translocation issue is intriguing since, as shown in this study, PBCV-1 host cells are packed with thylakoid membranes that surround most of the cell periphery and hence generate a formidable barrier for DNA translocation, as are all intracellular membrane structures [24].

To obtain insights into the initial events of the PBCV-1 infection cycle we used advanced super-resolution fluorescence and electron microscopy techniques, including Stochastic Optical Reconstruction Microscopy (STORM) that enables sub-diffraction resolution [25], Scanning-Transmission Electron Microscopy (STEM) tomography, and specific DNA labeling technologies such as Electron Microscopy *In Situ* Hybridization (EMISH). We demonstrate that shortly after attachment to the host cell wall, PBCV-1 perforates the wall and generates a membrane-lined tunnel through the fusion of viral membrane and host cytoplasmic membrane. Viral genomes are then ejected through this tunnel and rapidly translocated to the host nucleus, possibly through viral-induced perforations of thylakoid membranes. Previous studies revealed that at late infection stages PBCV-1 generates cytoplasmic organelles, termed viral factories, in which viral assembly takes place, a feature characteristic of many eukaryote-infecting dsDNA viruses [8,14,26–36]. These findings, along with those reported here, which underline the bacteriophage-like traits revealed by PBCV-1 at early infection stages, imply that PBCV-1 uniquely combines a bacteriophage-like infection mechanism during early infection stages with a eukaryotic-like infection pathway in its late replication stages.

## Results

### Viral membrane fusion with host membrane generates a portal for DNA delivery

**Fig 1:**
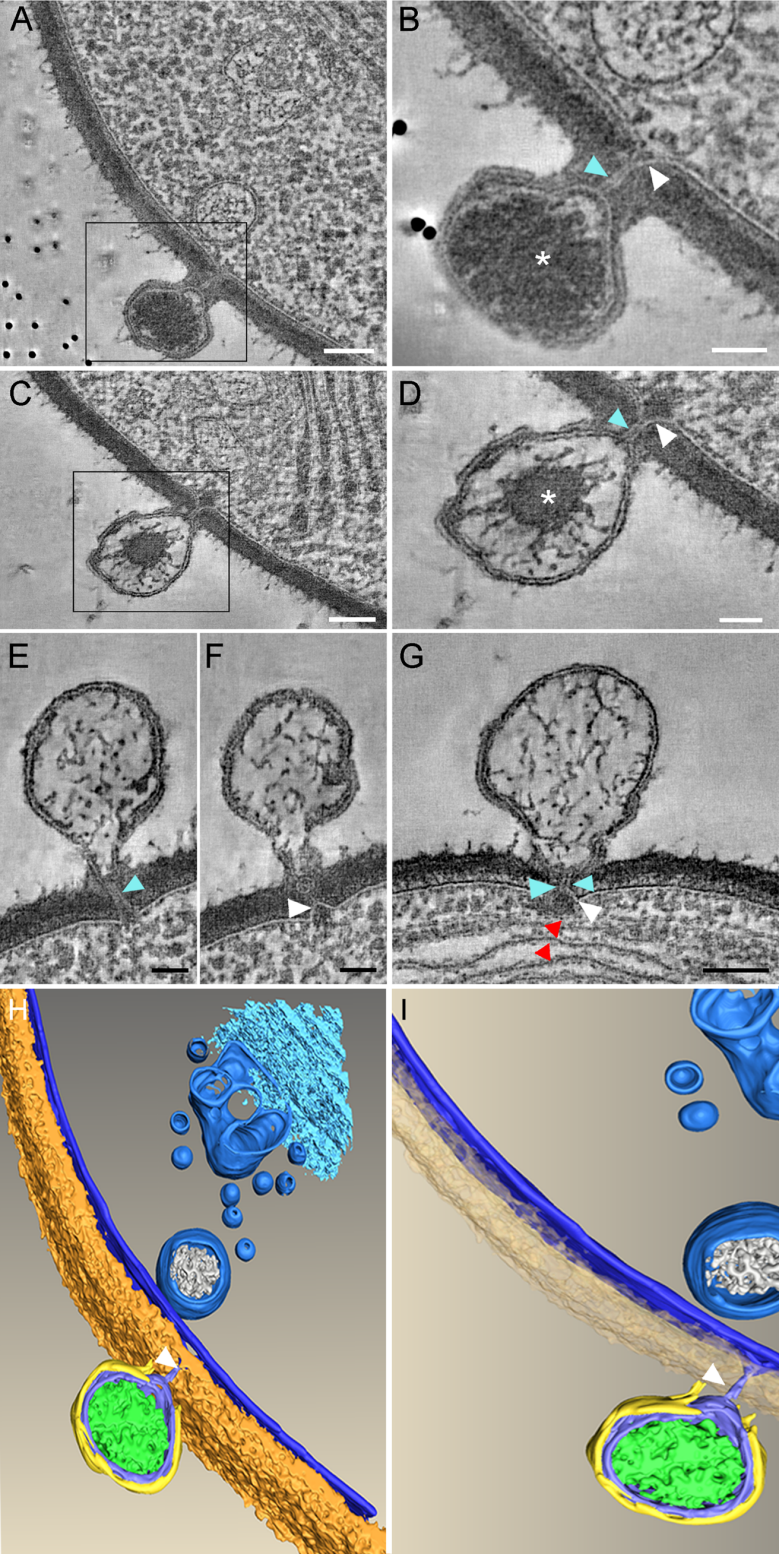
Host and viral membrane fusion generates a tunnel though which viral DNA is ejected. A-G. PBCV-1-infected chlorella cells at 1.5–2 min PI were immobilized with HPF-FS and thick sections were analyzed by STEM tomography. A. A 5.2 nm tomographic slice from a 220 nm-thick STEM tomogram showing the close proximity between the viral and host internal membranes resulting from their convergence at the infection site. B. A 7.8 nm tomographic slice of a high magnification of the inset in panel A. C. A 5.2 nm tomographic slice from a different 220 nm STEM tomogram. D. High magnification of the inset of panel C. The generation of a continuous tunnel is evident. E, F. Two different 5.2 nm STEM tomography slices from the same tomogram showing the same PBCV-1-infected cells with almost completely empty capsids in which the membrane tunnel is still detected. G. A 5.2 nm tomographic slice from a 216 nm-thick STEM tomogram exhibiting an empty capsid attached near thylakoid membrane stacks (red arrowheads). In all panels the membrane tunnel and the protrusion of the host membrane are marked with blue and white arrowheads, respectively. Asterisk: viral DNA. H, I. Volume rendering representation of the STEM tomogram shown in panel A. The 3D surface representation highlights the barriers that viral DNA has to overcome to reach the host nucleus (including cell wall, plasma membrane, cytoplasmic vesicles, Golgi, and photosynthetic membranes that were not captured in this tomogram). A PBCV-1 virion is attached to the cell wall (brown). The host membranes as well as cytoplasmic vesicles are marked in blue. The capsid is depicted in yellow, the internal viral membrane and the membrane tunnel (arrowheads) are shown in blue. Viral DNA is shown in green. Scale bars: A, C, G: 100 nm; B, D, E, F: 50 nm.

Previous studies of PBCV-1-infected chlorella cells demonstrated that shortly after attachment, PBCV-1 degrades the host cell wall and ejects its genome into the host cytoplasm [9]. To obtain deeper insights into viral DNA delivery, we used double-tilt Scanning Transmission Electron Microscopy (STEM) tomography of high-pressure-frozen and freeze-substituted (HPF-FS) PBCV-1-infected chlorella cells. Our tomography studies revealed that degradation of the cell wall at the virion attachment site is followed by the extension of the viral membrane towards the host cell (Fig 1). This deformation is accompanied by the protrusion of the host cellular membrane outwardly at the viral attachment site (Fig 1A–1G; white arrowheads), which is likely to result from the large turgor pressure within the host cells [10].

The concomitant deformation of viral and host membranes leads to a tight proximity between these membranes (Fig 1A and 1B), thus enabling fusion of the two membranes, which in turn results in the formation of a narrow membrane-lined tunnel of ~5 nm in its inner diameter and ~32 nm long (Fig 1, blue arrowheads). In addition, the PBCV-1 genome appears to undergo massive reorganization during its ejection (asterisks in Fig 1B and 1D), rearranging from an apparently homogenous morphology that is spread throughout most of the internal viral core (Fig 1A and 1B) to a mass that is positioned at the center of the capsid (Fig 1C and 1D). Upon completion of DNA ejection, empty capsids are left attached to the cell wall (Fig 1E–1G), frequently near multi-layered thylakoid membranes (Fig 1G; red arrowheads). In addition, the STEM tomograms revealed that the viral tunnel persists throughout the entire course of DNA delivery into the cell without changing its internal diameter. Our STEM data do not provide, however, an unambiguous answer on the fate of the tunnel following genome delivery.

Fig 1H, I shows a 3-D surface reconstruction derived from the STEM tomogram depicted in Fig 1A (S1 Movie). The infecting virus is attached to the cell wall (brown layer) and creates a hole in the wall, presumably using viral packaged enzymes. The tunnel generated following host and viral membrane protrusions and subsequent fusion is highlighted in Fig 1H and 1I (white arrowheads and blue structures, respectively).

### Viral genomes are rapidly transported to the host nucleus

**Fig 2:**
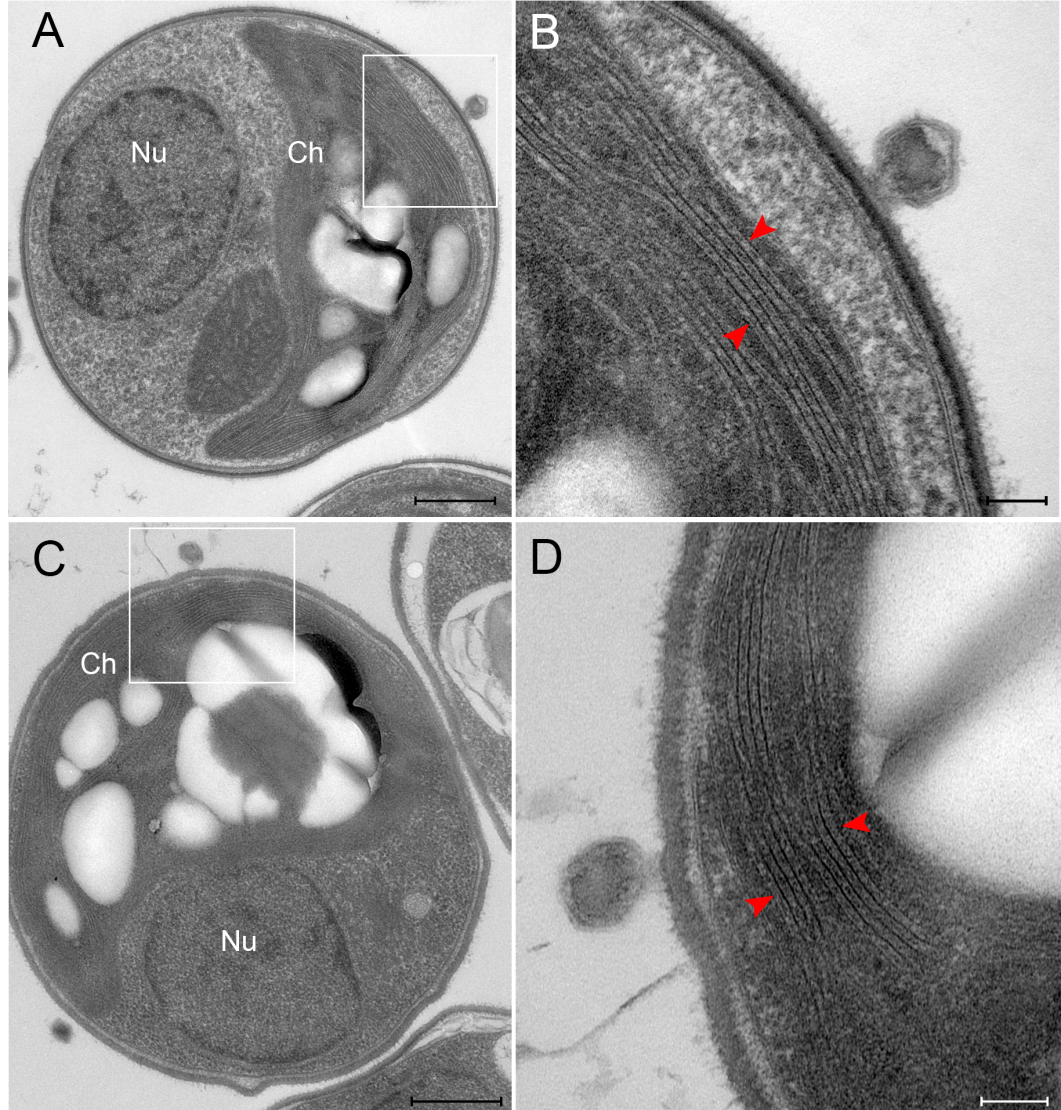
PBCV-1 infected chlorella cells at 2 min PI. A, C. TEM micrographs highlighting the hurdles that are associated with trafficking large viral DNA genomes to the nucleus. The large chloroplast is visible with an adjacent virus attached. The nucleus is located at the opposite side of the cell. B, D. High magnifications of panels A and C, respectively, showing the tightly packed thylakoid membrane (red arrowheads) and PBCV-1 virions attached adjacent to the thylakoid membrane stacks. Note that Panel D is rotated by 90o counter clockwise relative to Panel C. Scale bars: A, C: 500 nm; B, D: 100 nm.

Previous studies of the PBCV-1 infection cycle provided circumstantial evidence that following ejection of the viral genome into the host cytoplasm, PBCV-1 DNA and viral proteins are rapidly translocated towards and into the host nucleus [10,23]. Details on how this translocation occurs remain unknown. Specifically, the inherent hurdles associated with trafficking of the large PBCV-1 genomes through the crowded cytoplasm are highlighted in the STEM-derived model (Fig 1H and 1I, S1 Movie) that reveals numerous vesicles, cisternae and Golgi stacks in the host cytoplasm. The multiple, densely-packed chloroplast membrane stacks that surround most of the host cell (Fig 2) are likely to impose an additional and particularly demanding hurdle that viral DNA needs to overcome in its trajectory towards the host nucleus.

As indicated above, the internal diameter of the membrane tunnel generated by the fusion of the host and internal viral membranes is very narrow (~5 nm), presumably allowing concomitant transfer of only a single dsDNA helix along with putative viral DNA-binding proteins [22]. This result was unexpected given that the entire 331 kbp dsDNA PBCV-1 genome is translocated through the tunnel in only a couple of minutes. Such a rapid genome transfer is indicated by the finding that empty capsids attached to chlorella cells are detected already at two minutes following exposure of the cells to the PBCV-1 viruses (Fig 1E–1G).

**Fig 3:**
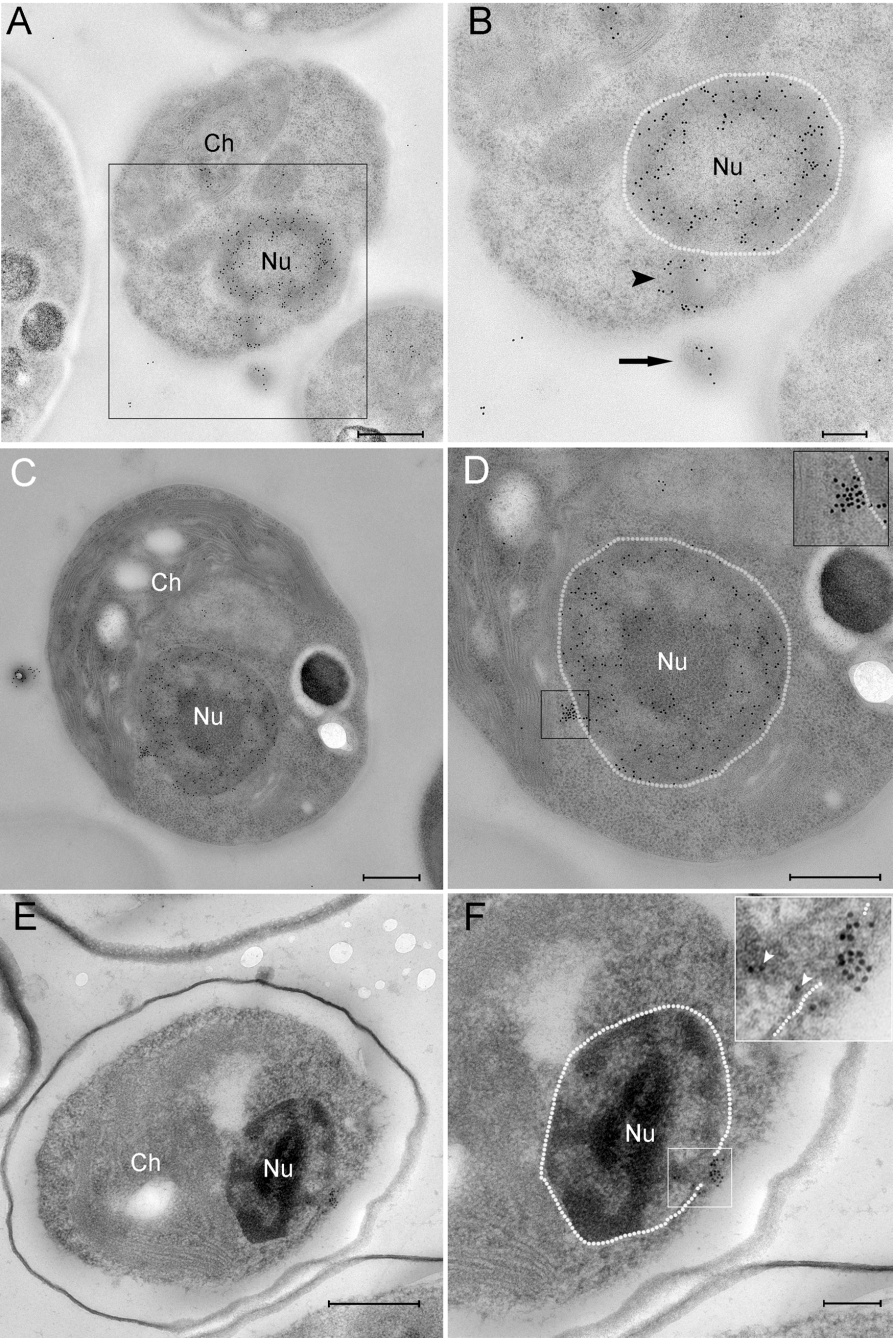
PBCV-1 genomes are transported towards the host nucleus. A-D. Cells infected with PBCV-1 for 2–6 min, immobilized using HPF-FS and thin sections were immuno-labeled with anti-DNA antibodies. A. Low magnification view of an infected cell. B. High magnification of the region indicated in panel A revealing a partially empty capsid (arrow) in the process of DNA ejection and translocation to the nucleus (arrowhead; nucleus contour is delineated with a dashed white line). Note that the viral DNA bypasses cytoplasmic barriers. C. Low magnification of a cell that was infected with PBCV-1 exhibiting dense DNA labeling close to the nucleus. D. High magnification of the cell in panel C. The dense and clustered DNA labeling near the nucleus strongly implies a condensed viral DNA morphology. Inset shows high magnification of the cytoplasmic DNA (nucleus borders are delineated with a dashed line). E, F. Cells were infected with PBCV-1 for 6 min and then chemically fixed and thin sections were subjected to EMISH. E. Low magnification of a cell illustrating dense viral DNA near the nucleus. F. High magnification view of panel E. Note that viral DNA is on a possible entry into the nucleus (white arrowheads in the inset). Nucleus contour is delineated with white dashed line. Nu: nucleus, Ch: chloroplast. Scale bars: A, C, D, E: 500 nm; B, F: 200 nm.

To localize viral genomes and follow their trajectories we used both immuno-DNA labeling of cryo-preserved specimens as well as Electron Microscopy In Situ Hybridization (EMISH) technology, which allows to specifically identify viral DNA. Briefly, EMISH methodology relies on hybridization of digoxygenin-labeled DNA probes with viral DNA sequences, followed by treatment with anti-digoxygenin antibodies. In addition to detecting cellular DNA localized in the host nucleus and chloroplast, anti-DNA antibodies revealed labeled DNA extending from infecting PBCV-1 particles (Fig 3A and 3B, black arrowhead), suggesting a viral genome in the process of being ejected into the cytoplasm and translocated towards the nucleus. Indeed, DNA was also detected near the host nucleus (Fig 3C and 3D and S1A and S1B Fig). Within 6 min, PBCV-1 genomes were detected in the vicinity of the nucleus and inside it (Fig 3E and 3F, S2 Fig). Significantly, this is the first visual evidence that PBCV-1 DNA actually enters the nucleus.

**Fig 4:**
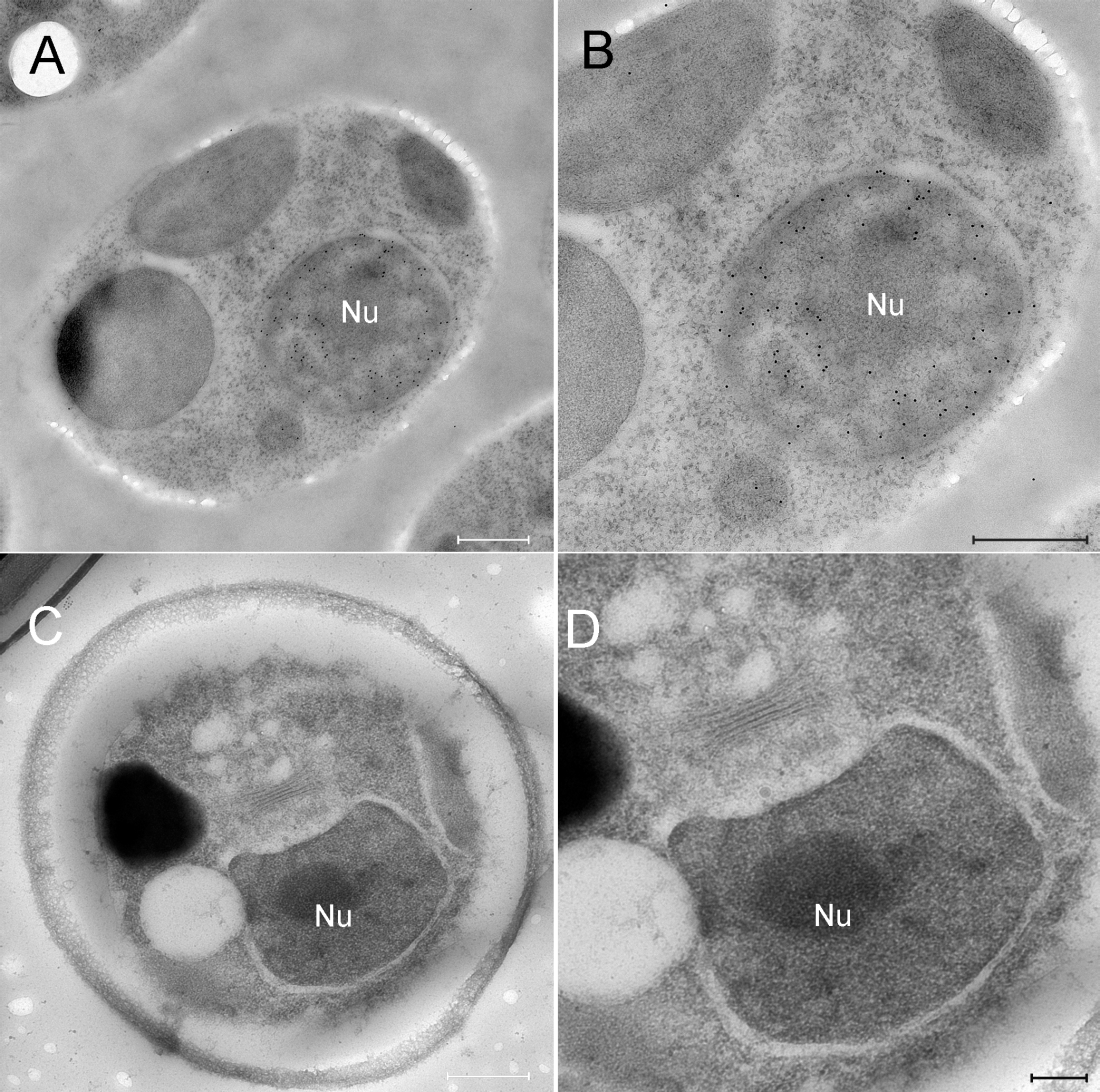
DNA immunolabling and in-situ hybridization of mock-infected chlorella cells. A, B. Mock-infected cells were cryo-immobilized and thin sections were immuno-labeled with anti-DNA antibodies. A. Low magnification view of a mock-infected chlorella cell. B. High magnification view of the cell shown in panel A. Whereas the nucleus exhibits dense DNA labeling, no cytoplasmic DNA labeling is detected. C, D. Mock-infected cells were processed for EMISH and thin sections were labeled with PBCV-1 specific DNA probes. C. Low magnification view of a mock-infected chlorella cell. D. High magnification view of the nucleus region revealing no DNA labeling, thus further validating the specificity of the viral DNA probes. Nu: nucleus. Scale bars: A, B, C: 500 nm; D: 200 nm.

The results depicted in Fig 3 imply that viral genomes are translocated as condensed structures rather than as extended, linear molecules (note the dense labeling of viral DNA in Fig 3D and 3F). Specifically, analysis of EMISH sections derived from 18 PBCV-1-infected chlorella cells revealed 16 cases of condensed DNA morphologies and two extended structures. In addition, general antiDNA immunoTEM studies of nine infected cells indicated seven cells with clearly condensed morphologies and two cells with in which the structure of viral genomes could not be precisely defined. Since slices used for EMISH and immunoTEM studies were obtained from random sectioning of infected cells at diverse cell volumes, these results strongly support the notion that viral genomes are translocated as condensed structures. This finding is consistent with the notion that condensed DNA conformations enable trafficking in the dense cytoplasm milieu by facilitating the bypass of cellular obstacles [8]. Notably, in immuno-DNA labeling assays, mock-infected cells revealed DNA labeling in the nucleus and chloroplasts, consistent with the presence of DNA in these organelles (Fig 4A and 4B), but no cytoplasmic DNA labeling. In addition, EMISH analysis of mock-infected cells hybridized with PBCV-1 DNA probes did not reveal any viral DNA sequences in the host cytoplasm or nuclei (Fig 4C and 4D). Further validation of the specificity of viral DNA probes was obtained with PCR and hybridization assays on thin transmission electron microscopy sections of mature virions (S3A and S3B Fig).

**Fig 5:**
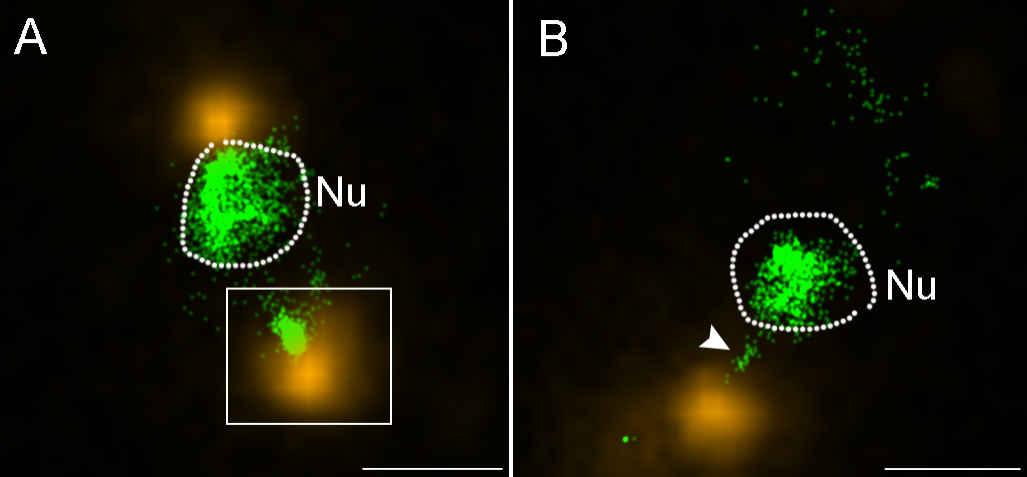
STORM analyses of PBCV-1 infected cells reveal condensed cytoplasmic viral DNA. Cells were infected for 1.5–2 min and processed for immuno-florescence with anti-PBCV-1 capsid antibodies, counterstained with SYTOX Orange for DNA detection and analyzed by STORM. A. A STORM image showing a host cell with two capsids at its periphery (yellow). Inset shows a virus delivering its genome (green) into the host cytoplasm at the opposite side of the nucleus. B. Another chlorella cell with an adjacent virus particle in the process of delivering its DNA into the nucleus (white arrowhead). In both panels the dashed line marks the nuclear boundary. Nu: nucleus. Scale bars: 1 mm.

Attempts to detect viral genomes inside infected cells using conventional fluorescence microscopy were unsuccessful due to diffraction resolution limit. Therefore, we used the Stochastic Optical Reconstruction Microscopy (STORM) technology that allows for the localization and identification of single-emitting fluorophores and reconstruction of high resolution images [25]. Our STORM studies consisted of immuno-labeling PBCV-1 capsids at 1.5–2 min PI with anti-capsid antibodies, followed by counterstaining with SYTOX Orange for DNA detection and localization. Fig 5A shows a capsid attached to the cell wall at the opposite side of the nucleus, and a condensed DNA structure extending from the capsid. Further STORM analyses of PBCV-1-infected chlorella cells reveal condensed viral DNA extending from capsids towards the nucleus (Fig 5B). It should be noted that the viral capsids depicted in Fig 5 are either empty or almost empty, as no DNA staining was detected. Altogether, the STORM results support our immuno-DNA labeling Electron Microscopy studies, which imply that, following ejection, viral genomes effectively overcome cellular obstacles in their trajectory towards the host nucleus, presumably by assuming a condensed morphology.

### Viral genomes are detected in photosynthetic membrane stacks

**Fig 6:**
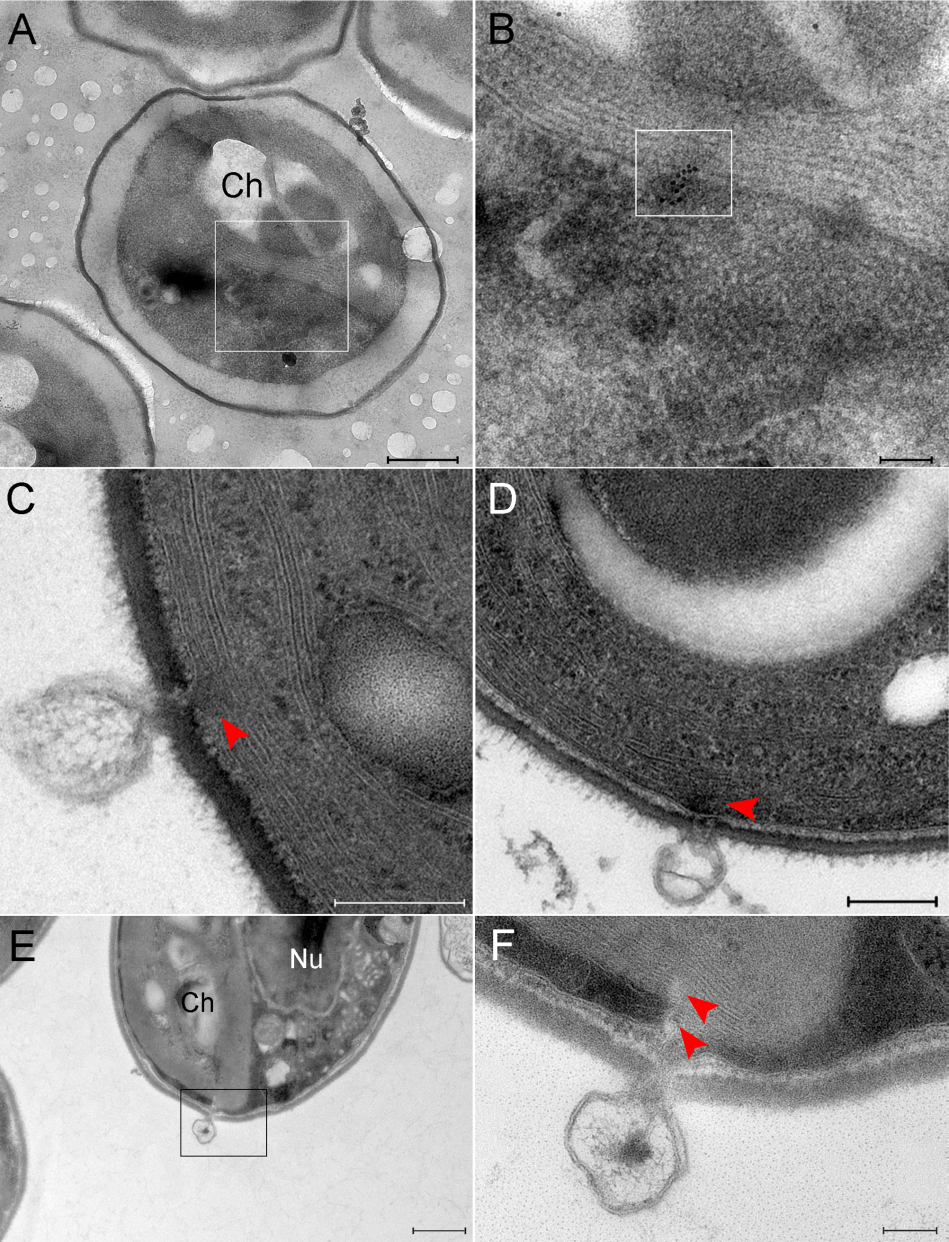
PBCV-1 genomes detected in host chloroplasts, presumably on a direct track to the host nucleus. A, B. Cells infected with PBCV-1 for 6 min and thin sections were subjected to In Situ hybridization. A. Low magnification view of a cell showing viral DNA inside chloroplasts. B. High magnification view of inset in panel A. Viral DNA is marked by the inset. Viral DNA is located at membrane stacks on a trajectory to the nucleus. C, D. TEM sections of PBCV-1 infected cells at 2 min PI. Empty capsids are detected near chloroplast stacks. Note the discontinuity of the chloroplast membrane stacks (red arrowhead) at the point of DNA ejection. E, F. Thin section of a 2 min PI chlorella-infected cell. E. Low magnification view of a chlorella cell with a virus near the chloroplast in the process of DNA ejection. F. High magnification view of the inset in panel E. Note the discontinuity of the thylakoid membrane stacks (red arrowheads).Nu: nucleus, Ch: chloroplast. Scale bars: A,E: 500 nm; B,F: 100 nm; C, D: 200 nm.

**Fig 7:**
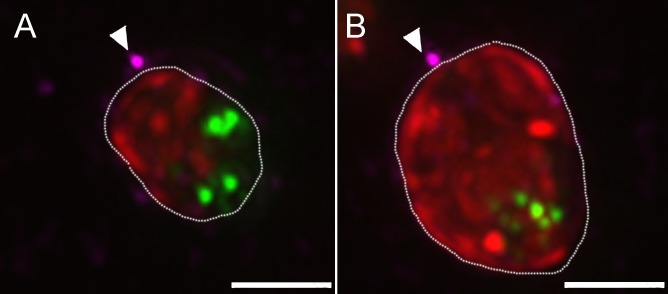
Representative projections of the entire volume of PBCV-1 infected cells. A, B. Views of the entire volume of de-convoluted fluorescence images showing 3h PI PBCV-1 infected cells stained for DNA (Sytox; green) capsids (anti-capsid antibody, magenta) and chloroplast auto-florescence (red). Arrows point to the possible infecting viruses near the chloroplasts. New viral progeny packed with DNA are represented by green dots in the cytoplasm of the cells, indicating successful infection. Scale bars: 2 μm.

Empty viral capsids attached near the host chloroplast are frequently observed. This observation underscores the question how do large viral genomes bypass the multilayered thylakoid membranes? An intriguing answer is provided by the observations that viral DNA is detected inside chloroplasts and that a discontinuity of the thylakoid membrane stacks is discerned at the point of viral DNA localization (Fig 6), implying either the use of preexisting gaps in the thylakoid membrane stacks or direct degradation of the membranes. As implied by TEM thin sections and STEM tomograms, the diameter of the perforations in the thylakoid membranes is ~5 nm. As our EMISH studies revealed viral genomes in the host chloroplasts, we conducted immuno-fluorescence assays at late PI time points to examine the notion that viruses attached to the cell wall next to chloroplasts are indeed capable of inserting their DNA through the multilayer thylakoid membranes (Fig 7). Our imaging observations, which comprise the entire volume of infected chlorella cells, demonstrate that even virions adsorbed near the host chloroplasts appear to generate viral factories. These virions are therefore infective, thus supporting the notion that viral genomes can be translocated through the thylakoid membrane stacks in their trajectory towards the host nucleus.

To exclude the notion that viral DNA can bypass the chloroplast to reach the nucleus, we carried out Focused Ion Beam Scanning Electron Microscopy (FIB-SEM) studies. This methodology enables capturing the entire volume of a chlorella cell at high resolution. S2 Movie reveals volume imaging of a mock-infected cell, demonstrating that the chloroplast occupies the entire volume, from top to bottom on one side of the cell, implying that a virus attached near the chloroplast must deliver its DNA through it, without the ability to bypass it in order to reach the nucleus. This finding supports the results of our immuno-fluorescence assays, suggesting that these viruses are infective (Fig 7).

## Discussion

**Fig 8:**
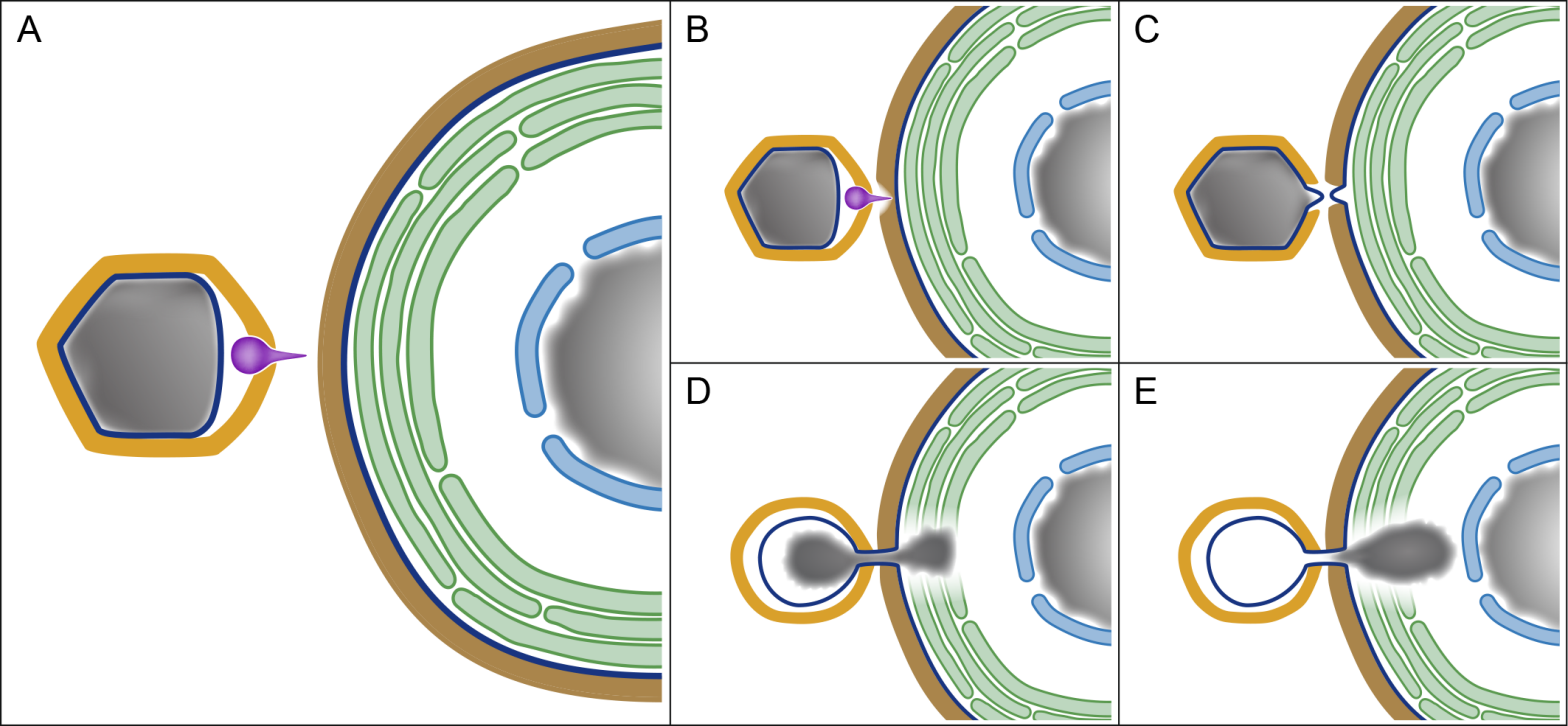
A model depicting early stages of PBCV-1 infection and highlighting the similarity between PBCV-1 infection and that of bacteriophages. A. The main components involved in PBCV-1 infection, including the PBCV-1 icosahedral capsid (yellow), internal membrane (dark blue), genome (gray) and spike (magenta) on the left side of the panel, as well as the cell wall (brown), cellular membrane (dark blue), thylakoid stacks (green), nuclear membrane (light blue) and nucleus (gray) of the host on the right side. B-D. Spike-mediated perforation of the host cell wall, protrusions of the viral and cellular internal membranes and their subsequent fusion into a membrane tunnel. D, E. Internalization of PBCV-1 genome into the host cytoplasm through a membrane tunnel, accompanied by the perforation of the photosynthetic thylakoid stacks and apparently promoted by viral DNA condensation. Significantly, perforation of the host cell wall, generation of a membrane portal and genome condensation represent crucial infection stages of bacteriophages (see text for details).

A model summarizing the early stages of PBCV-1 infection and highlighting the similar patterns of bacteriophage and PBCV-1 early infection cycles is depicted in Fig 8. We demonstrate that within a couple of minutes after exposing the unicellular photosynthetic chlorella cells to PBCV-1, the virus attaches to the host wall, using a spike located at a unique PBCV-1 icosahedral vertex [11] (Fig 8A), and digests the wall at the attachment site (Fig 8B). The spike complex is subsequently removed [12], thus creating an opening in the vertex and enabling the generation of a portal. The viral genome is then ejected into the host cytoplasm through a 32 nm-long tunnel that, as shown here, is generated by the fusion of the virus internal membrane with the host membrane (Fig 8C and 8D). Notably, such an infection process, including a spike-dependent viral-host attachment that is followed by the removal of the spike complex and formation of a membrane tube was reported for some bacteriophages, such as PRD1 that, like PBCV-1, contains an internal membrane [37,38].

We suggest that membrane fusion is promoted by the large internal pressure within the host cell (discussed below), which enables protrusion of the host cellular membrane towards the viral membrane through the virus-generated aperture in the host wall (Fig 8C). The inner diameter of the tunnel, which persists throughout the process of genome delivery, is ~5 nm. Such a narrow portal is intriguing as it enables transfer of only a single double-helix DNA at a time. This linear, base-pair by base-pair DNA translocation represents an additional feature characteristic of bacteriophage genome ejection that proceeds through a narrow ‘nanotube’ membrane [38,39]. Significantly, this process differs from genome release pathways of other members of large dsDNA viruses such as Mimivirus, which proceeds through a large portal that allows a concomitant release of the entire genome [32], or Vaccinia virus that similarly has a single-step release of the entire genome [40].

Genome ejection in many bacteriophages proceeds through a two-step process, whereby a first ‘push’ stage is promoted by the very high pressure generated by the tight genome packaging within capsids, which amounts to 60 atmospheres (atm) (~6 MPa) [4,20,41]. The second stage has been suggested to involve genome pulling mediated by several putative mechanisms, including transcription-based internalization and hydrodynamic effects [3,4,18,21,42,43].

Estimates of the internal pressure within capsids that are based on DNA packaging densities imply that although the pressure in PBCV-1 virions is lower than typical pressures in bacteriophages, it is substantial [22] and as such is likely to contribute to the internalization process of the PBCV-1 genome. An additional barrier to PBCV-1 genome ejection is the turgor pressure inside chlorella cells, which is higher than that of bacteria [44], suggesting that host internal pressure represents an additional barrier to PBCV-1 genome ejection. However, this hurdle is at least partially mitigated by the viral-encoded potassium ion channels (Kcv) located in the viral internal membrane [45]. Fusion of the virus membrane with the host plasma membrane (Fig 8C and 8D) results in a rapid depolarization of the host membrane, thus enabling efflux of ions and water out of the chlorella cells [46], thus reducing the host cell turgor pressure. Significantly, several studies demonstrated that the initial infection stages of diverse bacteriophages involve depolarization of the bacterial host membranes, leading to massive efflux of positively charged ions (mainly K^+^, presumably accompanied by the efflux of additional cations as well as of diverse anions that have not yet been characterized) and water molecules [3,37,47–49]. The findings that bacteriophages as well as the eukaryotic-infecting PBCV-1 utilize a membrane-depolarization pathway to overcome host turgor pressure further supports the notion of a bacteriophage-like process of PBCV-1 infection.

The finding that the viral genome maintains a condensed morphology located at the center of the virion (Fig 1C and 1D) is intriguing, as thermodynamic considerations would have predicted a genome conformation dispersed throughout the viral core. While the source of this unique structure remains unclear, we propose that the multiple and abundant DNA-binding proteins previously shown to be present in the PBCV-1 core (19) promote a condensed DNA morphology.

TEM immuno-labeling and super-resolution fluorescence studies reported here indicate that shortly after being released into the host cytoplasm, the PBCV-1 genome assumes a condensed morphology. Such condensation is implied by the dense and highly clustered DNA labeling that is depicted in Fig 3 and is characteristic of condensed, rather than of dispersed, morphologies, as indeed demonstrated by the heavy DNA labeling revealed by virions shown in this figure. This condensation, which is shown here for the first time to occur during genome internalization of a eukaryotic-infecting virus, presumably plays a crucial role in PBCV-1 infection. In addition to promoting genome internalization, a compact DNA morphology is likely to facilitate passage of the large PBCV-1 genome towards and into the host nucleus within the crowded host cytoplasm. Notably, DNA condensation was proposed to represent a significant pulling force during bacteriophage genome ejection [19,43,50,51].

Once released into the host cytoplasm, the PBCV-1 genome is rapidly translocated towards and into the host nucleus where it is replicated and subsequently released into a cytoplasmic factory where viral assembly occurs [14,52]. As shown in this study, chlorella cells are packed with chloroplasts containing thylakoid membranes that surround most of the cell periphery, underlining the question how do PBCV-1 genomes overcome this major hurdle during their trajectory towards the host nucleus. Our studies reveal that shortly after PBCV-1 infection, host thylakoid membranes are perforated, thus paving a pathway for the virus genome towards the host nucleus. Indeed, viral DNA sequences are present in the host thylakoid membranes (Fig 6). Significantly, a proteome study revealed that PBCV-1 packages two viral-encoded putative phospholipases [53]. It is tempting to speculate that PBCV-1 uses these phospholipases to perforate the thylakoid membranes in order to generate a trajectory towards the host nucleus. This notion is consistent with reports indicating that almost immediately after PBCV-1 infection, substantial reduction in photosynthesis occurs [54,55]. Notably, after ejecting their genome, empty PBCV-1 virions remain attached to the host wall, as is the case for bacteriophages.

Taken together, the results reported here reveal that the initial infection process of the chlorovirus PBCV-1 genome is remarkably similar to the process used by many tailed-bacteriophages yet differs from the process used by eukaryotic-infecting viruses that initiate infection through internalization of the entire virion or a substantial part of the particle. The PBCV-1 infection cycle proceeds through perforation of the host cell wall, cell plasma membrane and thylakoid membranes, thus overcoming the obstacle imposed by these cellular components on the translocation of viral DNA towards the nucleus. Previous studies established that at late infection stages, PBCV-1 generates cytoplasmic organelles, termed viral factories, where viral assembly takes place, a feature characteristic of many eukaryotic-infecting large dsDNA and (+)RNA viruses [14,15,27,30,31,34–36,56–58]. Thus, PBCV-1 uniquely combines a bacteriophage-like mechanism during its early infection stages with a eukaryotic-like virus infection pathway in its late replication stages.

## Materials and methods

### Cells, viruses and sample preparation

*Chlorella variabilis* NC64A cells were grown under continuous light and shaking on a modified Bold’s basal medium (MBBM) [54]. PBCV-1-infected as well as mock-infected cells were prepared for electron microscopy studies, including STEM tomography, FIB-SEM and immuno-electron microscopy [14]. Multiplicity of infection (MOI) was 10 in all experiments with the exception of the STORM studies in which it was 20 and immuno-flouresence studies in which it was 1.

### Electron microscopy *in situ* hybridization (EMISH)

#### I. Sample preparation

Chlorella infected and mock-infected cells were fixed using 4% paraformaldehyde and 0.5% glutaraldehyde (v/v) in MBBM for 2h at room temperature (RT) and washed with phosphate-buffered saline (PBS). Cells were centrifuged and pellets were embedded in 3.4% agar. Dehydration was carried out in ethanol followed by infiltration with increasing concentrations of the methacrylate-based embedding resin HM20 in dry ethanol. Resin was polymerized using 0.5% di-benzoyl peroxide at 70°C for 72h. Thin sections were mounted on pioloform-coated Nickel grids.

#### II. DNA extraction from PBCV-1 and chlorella cells

Purified viruses were treated with DNase (RNase-Free DNase, Qiagen) for 1h at 37°C to remove host-contaminating DNA. Viruses were centrifuged and pellets were resuspended in lysis buffer (10 mM Tris-HCl, pH 8.0, 0.1 mM EDTA, 0.4% SDS, 2 mM DTT, 200 μl phenol) containing glass beads, and vortexed for 3 min. Viral lysates were centrifuged and the soluble, DNA-containing phase was vortexed with phenol:chloroform (1:1); DNA was precipitated with EtOH from the aqueous phase. Pellets were washed with 70% ethanol, air-dried and re-suspended in water. For DNA extraction from chlorella cells, 300 ml of mock-infected cells (~1.3 X 10^7^ cells/ml) were centrifuged. Pellets were re-suspended in 10 mM Tris-HCl, pH 8.0, 0.1 mM EDTA. Cell lysis as well as further processing of algal DNA were carried out as described above for PBCV-1 genomes.

#### III. Determination of viral DNA purity and specificity

DNA extracted from PBCV-1 virions was subjected to PCR analysis to validate its purity from host DNA contaminants. PCR reactions were performed with initial DNA denaturation at 95°C for 2 min followed by 30 cycles of the following steps: denaturation at 95°C for 15 sec, annealing at 58°C for 45 sec, and 1 min at 72°C for primer elongation. Final elongation was carried out at 72°C for 7 min. S3 Fig (Panel A) shows PCR results demonstrating that viral DNA does not contain contaminating chlorella DNA. See Table 1 for primers used for PCR analyses.

**Table 1:** Primers used to determine viral DNA purity.

Gene	Forward Primers	Reverse Primers	PCR Products
α-tubulin (Cholrella)	5’AGATTGTGGACCTGTGCCTG	5’ACTCGGTGATGTCCACGTTC	549
Ankyrin (PBCV-1)	5’TTATGGTTATGATGCATGCG	5’CCATGGTCCTGTTCAAACAC	427

#### IV. Generation of viral specific DNA probes

After validating that viral DNA was free of cellular DNA it was treated with restriction enzymes XhoI, XbaI, and EcoRI (New England Biolabs) and subsequently purified with Qiagen Nucleotide Removal Kit (Qiagen). To generate DNA probes, 300 ng of digested DNA products were labeled using a DIG-DNA labeling kit (Roche) according to the supplier protocol.

#### V. EMISH studies

Thin sections of mock-infected cells and PBCV-1-infected cells were deposited on small drops of 100 μg/ml proteinase K (Sigma) in 20 mM Tris-HCl pH 7.9, 5 mM CaCl_2_ for 30 min at 37°C. Grids were washed 3 times in DDW and boiled for 3 min in 2xSSC in 70% formamide. Grids were transferred to cold hybridization solution containing 50% formamide, 10% dextran sulfate, 400 μg/ml salmon sperm DNA (Sigma) and 5.6 ng/μl PBCV-1 DNA probes. Hybridization was carried out for 16h at 37°C. Grids were washed with 2xSSC, incubated with 1% BSA in PBS for 20 min at RT and then with sheep anti digoxygenin antibody conjugated to 10 nm colloidal gold beads for 2h at RT. Grids were washed with PBS and post-stained with 2% uranyl acetate and Reynold’s lead citrate. Samples were visualized using FEI Spirit Tecnai T-12 and micrographs were recorded with an Eagle 2Kx2K FEI CCD camera (Eindhoven, the Netherlands).

#### VI. STEM tomography

Sections of ~250 nm were transferred to 150-mesh copper grids supported by carbon-coated (Edwards) Formvar film, and decorated with 12nm gold beads on both sides. Tilt series were acquired with FEI Tecnai G2 F20 TEM operated at 200 kV. Automatic sample tilting, focusing and image-shift correction were performed with Xplore3D software (FEI). Double tilt series were acquired at 1.5^0^ increments at an angular range of -65^0^ to +65^0^, with Gatan bright-field detector in the nanoprobe STEM mode. 3D reconstructions were computed from tilt series using a weighted back-projection algorithm. Tomograms were post processed either with a median or a smoothing filter using IMOD (See Ref. 33 for additional details).

### Generation of antibodies for the PBCV-1 major capsid protein Vp54

To detect PBCV-1 in immuno-fluorescence assays we raised antibodies against the major capsid protein, Vp54 using a short peptide sequence, NDDRYNYRRMTDC, derived from the full-length protein. The synthetic peptide conjugated to KLH (Keyhole Limpet Hemocyanin), a carrier protein extensively used to enhance antibody production on Cys residues was used to immunize 5 mice.

### Conventional immuno-fluorescence studies

Chlorella cells were infected with PBCV-1 at MOI of 1 for 3h and fixed with 4% paraformaldehyde (EMS) for 15 min at RT. Cells were washed in PBS and transferred to Poly-Lysine coated glass microwell dishes (Mat-Tek corp.). Cells were then blocked with 4% BSA-PBS solution for 30 min at RT and exposed to anti-capsid antibody diluted in 4% BSA-PBS for 1h at RT. Following washes in PBS, a secondary antibody, goat anti-mouse IgG conjugated to Alexa488 (Life Technologies) diluted in 4% BSA-PBS solution was added for 30–45 min. Cells were then counterstained with 500 nM SYTOX Orange (Life Technologies) in DDW for 15 min. Fluorescence images were visualized and photographed using a Deltavision system (Applied Precision) equipped with X100 UPlanSApo NA 1.40 objective. Fluorochromes were excited at 490 nm for Alexa488, 555 nm for SYTOX Orange and 640nm for chlorophyll (auto-fluorescence). Images were acquired with a photometrics coolSNAP HQ2 CCD (Roper Scientific) and de-convoluted with SoftWorx package using high noise filtering and 10 iterations. Image analysis and processing were conducted with Image J and Adobe Photoshop CS4-extended softwares.

### Super-resolution STORM imaging [25]

Chlorella cells were infected with PBCV-1 for 1–2 min at MOI of ~20 and fixed with 4% paraformaldehyde for 15 min at RT. Cells were washed with PBS and transferred to Poly-Lysine coated glass dishes (Mat-Tek corp.), blocked with 4% BSA-PBS solution for 30 min at RT and exposed to anti-capsid antibody for 1h. Following washes in PBS, goat anti-mouse IgG conjugated to Alexa488 (Life technologies) diluted in 4% BSA-PBS solution was added for 30 min for labeling viral capsids. Cells were washed in PBS and counter-stained with 5 nM SYTOX Orange (Life technologies) in DDW. Images were collected on a Vutara SR200 STORM microscope (Bruker). Before performing super resolution imaging, virus locations were identified by conventional fluorescence using Alexa488 labeling and 488 nm laser excitation (~5kW/cm^2^). DNA structures labeled with SYTOX Orange were imaged in super resolution using 561 nm laser excitation power of ~15kW/cm^2^. Images were recorded using a X60, NA 1.2 water immersion objective (Olympus) and Evolve 512 EMCCD camera (Photometrics). Data were analyzed with Vutara SRX software.

## Supporting information

S1 Fig General DNA immunolabling of PBCV-1 infected chlorella cells. (DOCX)

S2 Fig PBCV-1 genomes are rapidly transported towards the host nucleus. (DOCX)

S3 Fig Validation of the specificity of viral DNA probes used for EMISH. (DOCX)

S1 Movie 3D surface rendering of a PBCV-1 infected chlorella cell. Double-tilt STEM tomography analysis highlighting the hurdles associated with trafficking of large viral genomes in the highly crowded environment of the cytoplasm of chlorella cells. Slices derived from this tomogram are depicted in Fig 1. The movie was generated using Avizo (FEI).
(MP4)

S2 Movie FIB-SEM movie (Slice and View) of a mock-infected chlorella cell. The movie supports the immunofluorescence assay in Fig 7. Remarkably, the movie highlights the notion that viral DNA cannot by pass the chloroplast if a virus attaches adjacent to it. This is underscored by the fact that the chloroplast is tightly attached to the plasma membrane and occupies most of the cellular volume. The contrast of the images was inverted to match the TEM and STEM images. Each section is ~10 nm thick. The movie was created using image J.
(AVI)
