# Supplementary material for: Structural studies demonstrating a bacteriophage-like replication cycle of the eukaryote-infecting Paramecium bursaria chlorella virus-1
Source: PLoS Pathog. 2017 Aug 29;13(8):e1006562. doi: 10.1371/journal.ppat.1006562 (PMC5593192; doi:10.1371/journal.ppat.1006562)
Supplement: S1 Fig — (DOCX) [file ppat.1006562.s001.docx]

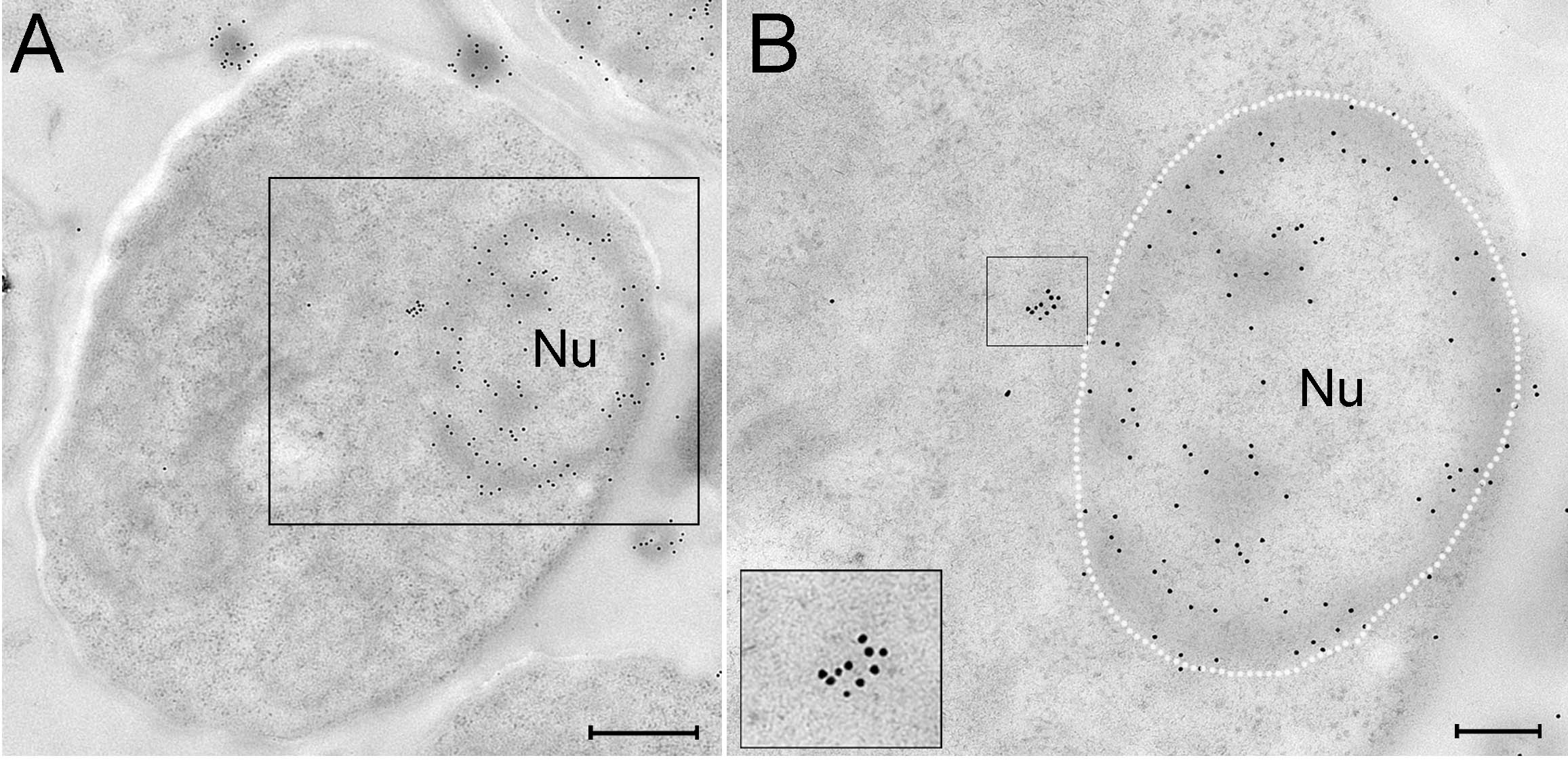


**Figure S1:** **General DNA Immunolabling of PBCV-1 Infected Chlorella Cells.**

**A, B.** PBCV-1-infected cells were cryo-immobilized using HPF-FS and thin sections were immuno-labeled with anti-DNA antibodies. **A.** Low magnification view of a 2-6 PI infected cell. **B.** High magnification of the region in panel A. Note the clustered DNA labeling that implies a condensed conformation of the viral DNA near the nucleus. Inset: high magnification of the apparently viral DNA (nucleus borders are delineated with a white dashed line). Nu: nucleus. Scale bars: A: 500 nm; B: 200 nm.
