# Supplementary material for: Structural studies demonstrating a bacteriophage-like replication cycle of the eukaryote-infecting Paramecium bursaria chlorella virus-1
Source: PLoS Pathog. 2017 Aug 29;13(8):e1006562. doi: 10.1371/journal.ppat.1006562 (PMC5593192; doi:10.1371/journal.ppat.1006562)
Supplement: S2 Fig — (DOCX) [file ppat.1006562.s002.docx]

**
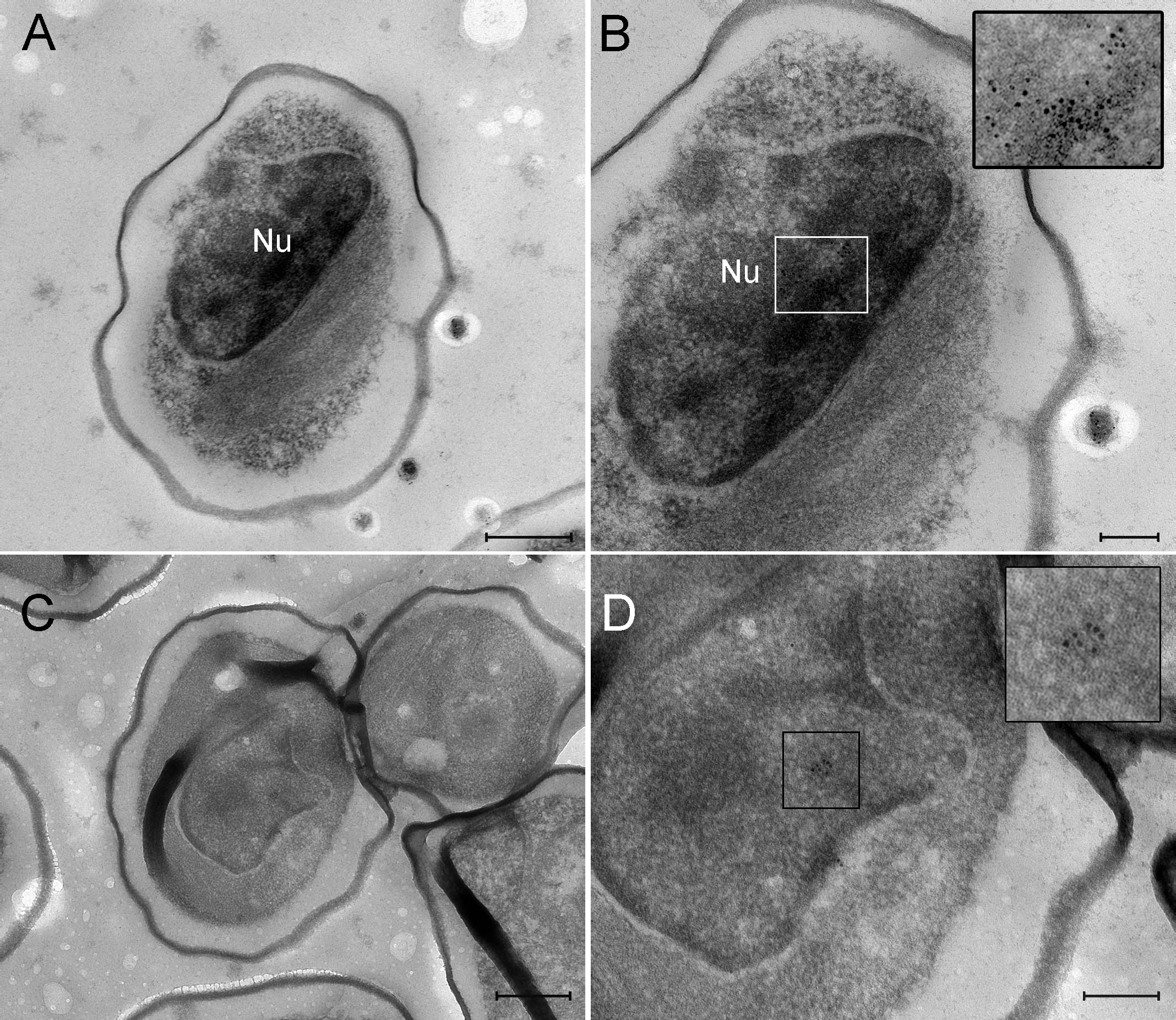
**

**Figure S2:** **PBCV-1 Genomes Are Rapidly Transported Towards The Host Nucleus.**

**A-D.** Cells were infected with PBCV-1 for 6 min and then chemically fixed and thin sections were subjected to EMISH. **A.** Low magnification of a cell illustrating dense viral DNA in the nucleus. **B.** High magnification view of the cell in panel A. Note that the viral DNA is inside the nucleus of the cell (inset – the brightness and contrast were adjusted to better visualize the viral DNA). **C.** Low magnification view of a different chlorella-infected cell showing PBCV-1 DNA inside the nucleus. **D.** High magnification view of the cell in panel C showing PBCV-1 DNA inside the nucleus (inset). Nu: nucleus. Scale bars: A, C: 500 nm; B, D: 200 nm.
