# Supplementary material for: Structural studies demonstrating a bacteriophage-like replication cycle of the eukaryote-infecting Paramecium bursaria chlorella virus-1
Source: PLoS Pathog. 2017 Aug 29;13(8):e1006562. doi: 10.1371/journal.ppat.1006562 (PMC5593192; doi:10.1371/journal.ppat.1006562)
Supplement: S3 Fig — (DOCX) [file ppat.1006562.s003.docx]

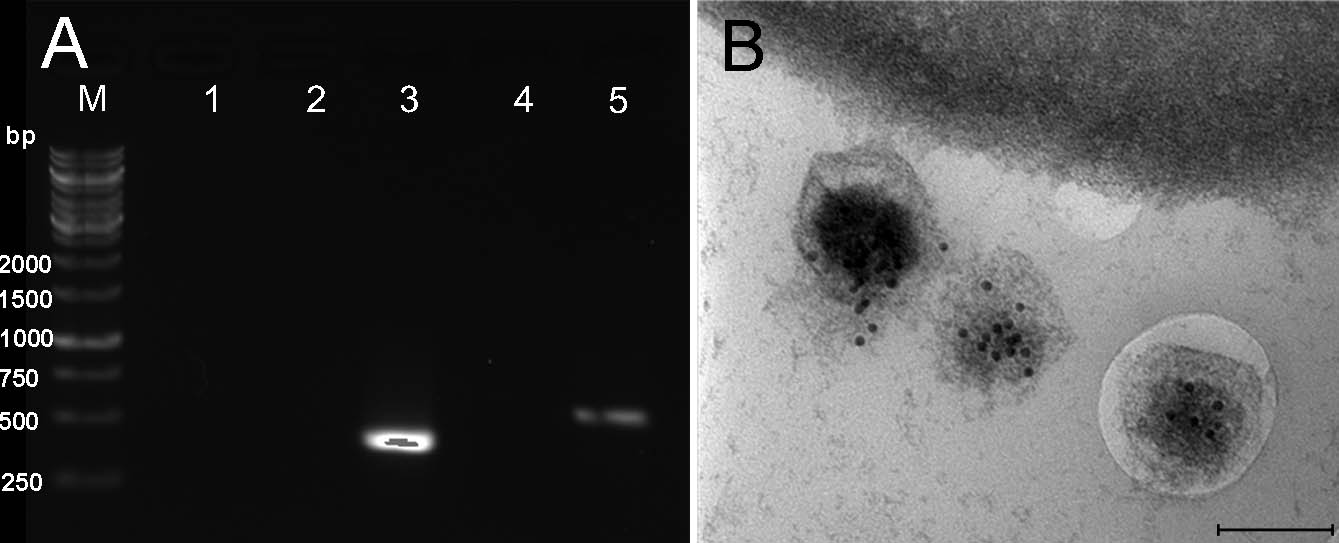


**Figure S3: Validation of the Specificity of Viral DNA Probes Used for** **EMISH.**

**A.** Chlorella and viral DNA were subjected to PCR. PCR products were separated by DNA gel electrophoresis. Lanes: **1,2**: PCR with primers for tubulin and ankyrin with no DNA templates of (1) Chlorella cells and (2)_PBCV-1. **3**: Viral DNA subjected to PCR with ankyrin primers. The 427bp product validates that the viral DNA is present and amplified as expected. **4**: Viral DNA subjected to PCR reaction with Chlorella alpha tubulin primers. No bands are detected, thus demonstrating that viral DNA is pure with no host DNA contaminations. **5**: Chlorella DNA subjected to PCR with alpha tubulin primers. As expected the 549bp product is visible. M- marker. **B.** Cells were infected with PBCV-1 for 6 minutes and then chemically-fixed and thin sections were used for *In Situ* hybridization. A high magnification view of extracellular viruses exhibiting dense DNA labeling at their core. This representative image confirms that the viral DNA probes are highly specific for PBCV-1 DNA. Scale bar: 100nm.
